# Impact of Bleaching before or after Veneer Preparation on Color Masking Ability of Laminate Veneers: An In Vitro Study

**DOI:** 10.1155/2021/6611173

**Published:** 2021-04-27

**Authors:** Gollshang Ahmad Mhammed Dalloo, Bestoon Mohammed Faraj, Abdulsalam Rasheed Al-Zahawi

**Affiliations:** Conservative Department, College of Dentistry, University of Sulaimani, Iraq

## Abstract

**Purpose:**

This study evaluates the effect of bleaching before or after veneer preparation and the depth of preparation on color masking ability of laminate veneers*. Methods*. Sixty extracted premolars were artificially stained to vita shade A4, verified by digital spectrophotometer (Vita Easy Shade V), and then divided into three groups: NB = nonbleached, BBP = bleaching before preparation, and BAP = bleaching after preparation. Based on the preparation depths, each group was further divided into two subgroups: S1 = 0.5 mm and S2 = 1.0 mm. BBP and BAP were subjected to one session of in-office bleaching using 35% hydrogen peroxide. IPS e-max CAD veneers of 0.5 and 1.0 mm thickness (corresponding to the preparation depths) of the same shade and translucency (HT A1) were cemented immediately to the bleached surfaces. Immediately after cementation, the color change Δ*E* between the baseline (after staining) and the resulted shades was measured using the Vita Easy Shade V digital spectrophotometer and CIELab color system.

**Results:**

Bleached groups exhibited a significant Δ*E* value compared to the nonbleached group (*p* < 0.05). BAP showed the highest Δ*E* value. No significant difference was found between BBP and BAP. S2 revealed a significant Δ*E* value than S1 (*p* < 0.05). No significant difference was found between S1of BAP and S2 of NB, BBP, and BAP (*p* > 0.05). Regarding the color coordinates, the difference between the tested groups was highly significant in lightness (ΔL∗) (*p* < 0.001), while no significant differences were found in green/red value (Δ*a*∗) and yellow/blue value (Δ*b*∗) (*p* > 0.05).

**Conclusions:**

In cases of severe tooth discoloration, one session of in-office bleaching before or after veneer preparation and the preparation depth do not influence the color masking ability of laminate veneers.

## 1. Introduction

The aim of advanced dentistry is the preservation of the greatest tooth structure with maximum esthetics in a brief period [1]. Tooth discoloration is the cause of the most esthetic problems. This has induced patients and dentists to spend significant amounts of time and money to achieve an esthetically pleasing smile. Tooth discolorations are generally classified as extrinsic or intrinsic. Extrinsic tooth staining is associated with deposition of chromogens from outside the tooth structure, while intrinsic staining occurs due to structural or compositional changes or thickening of dentinal hard tissue [2]. Depending on the type and severity of tooth discoloration, the discolored tooth can be treated by tooth bleaching, veneering, or a combination of both bleaching and veneering. Even though at-home bleaching is a widespread practice and has a high rate of success and satisfactory results [3], there are still some people that prefer not to use bleaching trays or who require faster results. In addition, some patients may not adapt well to the daily use of a bleaching tray, which increases the treatment time and costs. In these circumstances, in-office bleaching seems to be an appropriate alternative to home bleaching for patients whose time is crucial and who desire faster results [4] and who do not want to wait for two to three weeks to see their results in cases of very severe discoloration and single tooth discoloration. In-office bleaching often requires more than one visit to get an optimal result. However, the final results may be unpredictable [1]. In severely discolored teeth that are not responsive to routine vital bleaching, it may be necessary either to extend bleaching time [5] or to reduce the enamel surface for veneering without exposing underlying dentine to encourage penetration of bleaching agents into the stained dentine [6, 7]. In attempting to treat severely discolored teeth by veneering, there is a considerable risk that the dark shade of the tooth tissue substrate will shine through relatively thin and translucent ceramics. Therefore, several approaches have been used to mask the underlying color; these include employing more opaque ceramics and luting cement [8–10], increasing the preparation depth [11] to allow adequate space for the ceramic thickness [12], or manipulation of the prepared tooth to lighten its shade [13]. Each of these approaches has its problems. Opaque ceramics and luting cement can often result in an opaque lifeless appearance. When cutting deeper into the tooth structure in order to allow more thickness of the ceramic, this may lead to the exposure of the dentine, which provides a weaker bond and is associated with a higher risk of failure [14]; it is therefore recommended that the design of preparation must be within the enamel layer as much as possible to achieve greater adhesion. It has been accepted that the ideal veneer preparation depth is 0.3 mm at the cervical margin, 0.5 mm for the middle third, and 0.7 mm on the incisal third [15]. Also, with cutting deeper into the discolored dentine and increasing the depth of preparation, the discoloration becomes darker. Therefore, a potential combination of bleaching and veneering to produce a veneer restoration with a lighter color has been demonstrated in some in vitro studies, with varying degrees of success [16–18].

To the extent of the authors' knowledge, the effect of in-office bleaching before and after enamel reduction on the final color of veneer restoration in one session, using human teeth in vitro, has not been investigated yet. Thus, this study is aimed at comparing the effect of one session of in-office bleaching and the depth of preparation on the final color of IPS e-max CAD veneer restoration in severely discolored teeth.

The first null hypothesis was that one session of light activated in-office bleaching before cementation of ceramic veneer does not affect the veneer restoration's final color. The second null hypothesis was that one session of light activated in-office bleaching after surface reduction has no effect on reducing the depth of preparation.

## 2. Materials and Methods

### 2.1. Study Design

**Figure 1:**
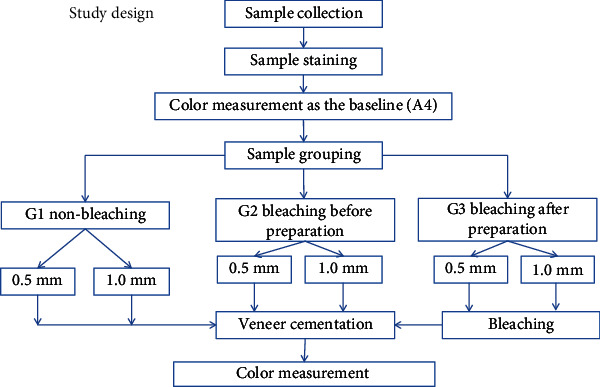
Summary of the study design.

Sixty premolar teeth, recently extracted for orthodontic purposes, were stained to A4 shade and then divided into three groups (*n* = 20). Group one (nonbleaching) was to act as the control group, and the other two groups were bleached using 35% hydrogen peroxide: group 2 was bleached before preparation for veneer restoration, and group 3 was bleached after preparation. Each of these three groups was subdivided into two subgroups (*n* = 10) of 0.5 mm and 1 mm according to the depth of preparation and veneer thickness (Figure 1).

The sample size was determined based on previous studies and their results, which conducted similar investigations and used the same sample size, with consideration of *α* = 0.05 and *β* = 0.1 [12, 19, 20].

### 2.2. Sample Collection

Sixty maxillary premolar teeth of similar size, extracted within the last three months for orthodontic purpose, were collected from dental clinics in Sulaymaniyah region, Iraq. The teeth were examined for any caries, cracks, or any other defects, then scaled and cleaned with pumice and rubber cup in a slow-speed handpiece. When not in use, the teeth were stored in distilled water to prevent dehydration. Any tooth with buccal surface dimensions of less than 8 mm was excluded from the study to allow adequate surface for color measurement.

To measure the thickness of the dental structure on the buccal surface of the teeth (for standardization), digital X-ray radiographs were taken by Vatech sensor size of 1.5 with an exposure time of 0.4 seconds and a 30 cm focus-object distance (70 kVp and seven mA). The external X-ray beam was focused at a 90-degree angle to the mesial surface of the tooth, and the buccal tooth thickness was measured with the EasyDent 4 viewer version 4.1.5.9 software [21].

The distance from the midpoint on the buccal surface to the top of the pulp horn was measured [22] as a straight line extending from the enamel surface to the DEJ (dentinoenamel junction) and from the enamel surface to the top of the buccal pulp horn, as the color measurements would be taken from the middle third of the buccal surface, by holding the tip of the spectrophotometer perpendicular to it. Based on these results, the total enamel/dentine thickness of the teeth was 4.00 ± 0.5 mm (enamel = 1.4 ± 0.2 mm; dentine = 3.6 ± 0.3).

### 2.3. Sample Preparation and Staining

The teeth were placed in 1% sodium hypochlorite for 30 minutes to remove any initial extrinsic stain. The roots were removed 2 mm apical to the cementoenamel junction under water cooling using a double-faced diamond disc (ISO 806, 91 1HF, Germany) attached to a slow speed handpiece, the pulp was then removed, and the chambers were cleaned. The dentine was etched with 37% phosphoric acid etching gel for 60 seconds by injecting the etchant into the pulp chamber (37% wt phosphoric acid, Super Etch, SDI). The etching gel was then removed by rinsing in water for a further 30 seconds. This was carried out to remove the smear layer and open the dentinal tubules to encourage stain uptake into the tooth [17].

The teeth were stained with a standardized tea solution using the method proposed by Sulieman et al. [16, 17]. The tea solution was prepared by boiling 2 g of tea (Marks and Spencer's Extra Strong tea, Marks and Spencer, London, UK) in 100 ml of distilled water for 5 minutes and then filtered through gauze to remove the tea from the infusion. Each specimen was immersed in 5 ml of the tea solution at room temperature (22 ± 2°C) in sealed universal containers, and the solution was renewed every day. This method results in a combination of both intrinsic and extrinsic staining, most of which is intrinsic. The stain development was monitored daily until shade A4 was obtained; before measuring the color of the specimens, they were polished with pumice for 30 seconds [1] using a bristle brush in a slow-speed handpiece to control the extrinsic stains. Throughout the study, all specimens were maintained in a fully hydrated environment in a sealed container.

### 2.4. Color Measurement

A standardized protocol for color evaluation was adopted for all the specimens by embedding each tooth in a special mold that allowed the buccal surface of the crown of the tooth to be exposed and providing a black background for color measurement. Vita Easy Shade V (Vita, Zahnfabrik, Germany), the new fifth generation of Easy Shade, was used in this study to measure the color changes. This is a more innovative and precise device which, due to the LED technology, is unaffected by ambient conditions and produces objective and reliable measurements. The probe tip illumination diameter is 5 mm. Software version v507k (serial number: H55328, light source: white LED D65) was used.

**Figure 2:**
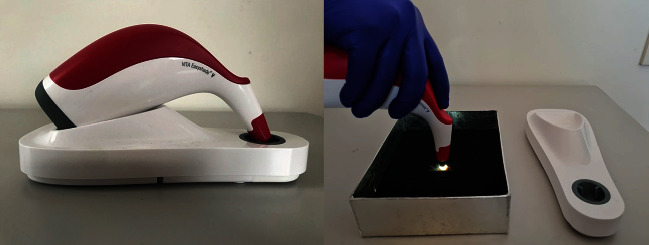
Tooth color measurement using Vita Easy Shade V Spectrophotometer.

Following the manufacturer's instruction, before measuring the tooth's color with the VITA Easy Shade V on the base station, the measurement button was used to run calibration. The tip of the probe was held and placed in contact with the tooth surface, and the angle of measurement was kept constant for all teeth by placing the tip of the probe perpendicular to the middle third area of the tooth surface (Figure 2). The color measurements were repeated three times, and the average value was used. To measure tooth color, CIEL∗a∗b∗ color system was used; the overall color change of each specimen (Δ*E*) was calculated for all the groups by applying the following equation:

(1) $$\begin{array}{c} \Delta E={\left[{\left(L2–L1\right)}^{2}+{\left(a2–a1\right)}^{2}+{\left(b2–b1\right)}^{2}\right]}^{0.5} \end{array}$$

where Δ*E* is the overall color change; *L*1, *a*1, and *b*1 are the baseline (after staining) color coordinate measurements; and *L*2, *a*2, and *b*2 are the color coordinate measurements after veneer cementation.

The experiment consisted of two phases of color measurement:

1. After staining as baseline
2. After veneer cementation

### 2.5. Sample Grouping

Following staining, the samples were randomly divided into three groups (*n* = 20):

1. G1: nonbleached (NB) group
2. G2: bleaching before preparation (BBP)
3. G3: bleaching after preparation (BAP)

Each group was then further divided into two subgroups (*n* = 10) according to the depth of preparation and veneer thickness:

1. 0.5 mm (S1)
2. 1.0 mm (S2)

### 2.6. Tooth Preparation

The same operator prepared all the teeth; a polyvinyl siloxane (silicone index) was used to ensure tooth reduction and control the depth of preparation.

**Figure 3:**
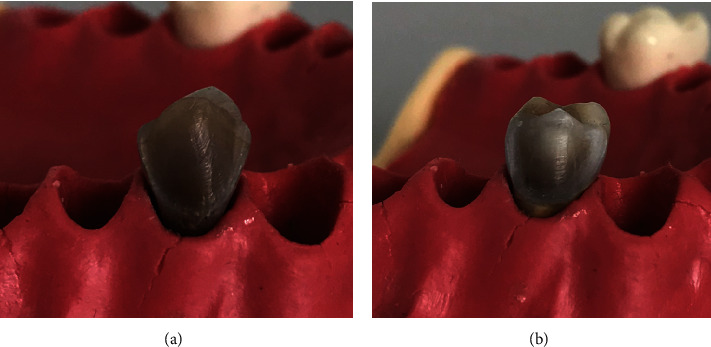
Preparation depths: (a) 0.5 mm and (b) 1.0 mm.

To approximate the clinical situation, all the teeth were mounted individually in a student typodont manikin (Frasaco, Tettnang, Germany) during the tooth preparation. Standardized veneer preparations were performed on the facial surface of the teeth at two different depths: 0.5 mm and 1.0 mm (Figure 3), for each subgroup. The occlusal finish line was prepared to a feather design, the preparation was terminated in the occlusal margin without shortening the cusp, and the cervical margin was placed about 1.0 mm coronal to the cementoenamel junction; proximally, the preparation extended just buccal to the contact area with a chamfer finish line. The facial surface of the tooth was initially prepared by making a cervical groove with a round end diamond bur (ISO 001-010, BR-45, Japan) to place the cervical and proximal margins at the correct depth and position, with the bur shaft held in contact with the facial surface. The depth-orientation grooves (0.5 mm/subgroup 1 and 1.0 mm/subgroup 2) were then placed with a self-limiting depth-cutting bur (552-018 M, VP-21 dental FG diamond veneer preparation bur and 552-021 M, VP-22 dental FG diamond veneer preparation bur). The bottoms of the grooves were marked with a permanent marker in order to determine the level to which the tooth structure should be removed. The unprepared enamel islands were then removed to the level of the horizontal depth orientation grooves, until the color marking had disappeared and without exceeding the depth orientation grooves, using a rounded end cylinder diamond bur (ISO 138-010 SR 41, Japan) to maintain a uniform depth of cutting. This reduction was performed by following the facial contour of the tooth.

A round-end diamond tapered fissure bur (ISO 197-016 TR-25, MANI, Japan) was used to place a chamfer finishing line and refine the preparation. Then, the prepared samples were additionally smoothed with a fine-grit round end diamond tapered fissure bur (ISO 198-018 TR-13F, Japan). The required time for each tooth preparation was about 10 minutes, and burs were replaced after every third tooth preparation. All tooth preparations were completed using compound loups (Rose Microsolution, USA) under ×2.5 magnification, without any sharp line angles, with a high-speed handpiece under water coolant. To ensure the reproducibility of the test specimens, several control measurements were done during preparation of the tooth by replacing it into the individual silicone index and checking for proper reduction of the buccal surface, from a lateral view.

### 2.7. Tooth Whitening

Power bleaching with Quick White bleaching agent (Quick White, British) was used to whiten the teeth in the BBP and BAP groups by mixing 0.1 g of the bleaching powder, which contains fumed silica, photoactive colorants, and blue dyes, with 0.5 ml of 35% hydrogen peroxide gel [16]. The hydrogen peroxide was heated in hot water (90°C) for 1-3 minutes before mixing with the powder, following the manufacturer's instructions. A layer of 2-3 mm thick of the mixture was applied to the tooth surface with a plastic spatula and activated for 30 seconds with a halogen light-curing unit (2500, 3M/ESPE, Germany) with a power density of 500-600 mW/cm^2^, placed just above the surface of the gel. The gel was left on the surface of the tooth for 10 minutes. The gel was refreshed by removing it with a damp piece of gauze and applying another layer to the tooth surface and further activated for 30 seconds and left for a further 10 minutes. This cycle was then repeated once more to complete the three, 10 minutes passing, according to manufacturer's instructions for the clinical use of the product.

### 2.8. Fabrication of Ceramic Veneers

All the restorations were fabricated from IPS e-max CAD for CEREC/inlab (IPS e-max CAD/Ivoclar Vivadent HT/A1, USA) blocks. The prepared teeth were scanned by a scanner (CEREC AC, Sirona, Dentsply/Germany) to obtain a 3D image of the preparation. These data were transmitted to a software program (CEREC SW 4.6.1.152739) to design the veneers. Next, the design was sent to the CAM nesting for milling by (CEREC MC XL, Sirona, Dentsply, Germany) milling unit. After milling, the veneers were ultrasonically cleaned in distilled water for 10 minutes and dried. A digital caliper was used to check the thickness from four points to ensure the final thickness (0.5 ± 0.02 and 1.0 ± 0.02) before they were coated on one side with a layer of neutral/shade glaze (Ivoclar Vivadent, Amherst, NY, USA) and crystallized using the manufacturer's instructions. Crystallization was carried out in a furnace (CEREC SpeedFire, Sirona, Dentsply, Germany), fired at 300°C/min for 10-15 minutes. The completed ceramic specimens were ultrasonically cleaned in distilled water before cementation.

### 2.9. Veneer Cementation

All the teeth were cleaned and polished with a pumice and rubber cup in a slow-speed handpiece; then, the prepared surface of the tooth was etched with 37% phosphoric acid for 15 seconds (37% wt phosphoric acid, Super Etch, SDI), rinsed with air-water spray for 15 seconds, and gently air-dried. Prime and bond universal™ (Dentsply Sirona De-Trey, Konstanz, Germany) universal adhesive was applied to the tooth surface with a clean disposable brush and slightly agitated for 20 seconds, air-dried for 5 seconds to evaporate the solvent, and then light polymerized with an LED light-curing unit (Elipar Light Cure, 3 M, ESPS), with an intensity of 1200 mW/cm^2^ for 10 seconds, according to the manufacturer's instructions.

The ceramic veneers were etched with 9.6% hydrofluoric acid gel (Pulpdent Corporation, Watertown, USA) for 60 seconds, then rinsed with water-air spray for 60 seconds and air-dried. After etching, the veneers were ultrasonically cleaned in distilled water for 5 minutes. A silane-coupling agent (Pulpdent Corporation, Watertown, USA) was applied with a clean brush in one layer and allowed to set for 60 seconds. Cementation of the veneers was performed according to the manufacturer's instructions using translucent Calibra veneer resin cement (Dentsply, Caulk, USA). The Calibra cement base paste was dispensed from the syringe directly onto the bonding surface of the veneer before seating it in place in its respective area on the prepared tooth, and a static load of 200 g was applied to the veneer before light curing. The excess cement was removed with a microbrush, and curing light was applied briefly for 10 seconds to tack the restoration in place. Any excess cement from the restoration margins was removed with a sharp instrument. Polymerization was conducted on all surfaces for 30 seconds using the LED light-curing unit (Elipar Light Cure, 3 M, ESPS, Germany), with an intensity of 1200 mW/cm^2^. Immediately after cementation, further color readings were performed by a Vita Easy Shade V spectrophotometer.

### 2.10. Statistical Analysis

After testing for normal distribution using the Shapiro-Wilk test, the statistical analysis was performed using SPSS statistical software version 25 for Windows (SPSS, Chicago, II, USA), and the level of confidence was set at *p* < 0.05. Analysis of variance (ANOVA) was used to analyze the data for significant differences. Post hoc Tukey's test was used to perform multiple comparisons among the groups.

## 3. Results

The mean and standard deviation for the change in color (Δ*E*) and color coordinates of lightness (Δ*L*∗), red/green value (Δ*a*∗), and yellow/blue value (Δ*b*∗) for the three tested groups (NB, BBP, and BAP) at two different depths of preparation and veneer thicknesses (0.5 mm (S1) and 1.0 mm (S2)) are presented in (Table 1).

The significance of the differences among the groups is demonstrated in (Table 2), using the ANOVA test.

The ANOVA test revealed a significant change in color (Δ*E*) among the three tested groups (NB, BBP, and BAP) (*p* < 0.05) (Table 2).

However, for both S1and S2, Tukey's HSD test revealed no statistically significant difference in Δ*E* among the three tested groups (NB, BBP, and BAP) (*p* > 0.05) (Table 1), and between the S1 and S2 for each tested group (*p* > 0.05), the statistically significant difference was between S2 of BAP group and S1 of BBP and NB groups (*p* < 0.05) (Table 1).

Regarding the spectrophotometer results, there was a highly significant statistical difference among all tested groups in Δ*L*∗ (*p* < 0.001) (Table 2). Tukey's test revealed that for S1 there was a significant difference between the NB and BBP and BAP groups (*p* < 0.05), while no significant difference was found statistically between the BBP and BAP groups (*p* > 0.05). For S2, Tukey's test of Δ*L*∗ identified no significant difference between the NB and BBP groups (*p* > 0.05), a highly significant difference between the NB and BAP groups (*p* < 0.001), and a statistically significant difference between the BBP and BAP groups (*p* < 0.05). In the NB and BBP groups, no statistically significant difference was found between S1 and S2 (*p* > 0.05), but there was a significant difference in the BAP group between S1 and S2 (*p* < 0.05) (Table 1).

The ANOVA test revealed no statistically significant difference in Δ*a*∗ and Δ*b*∗ (*p* > 0.05) among the three tested groups and between S1 and S2 of each tested group (Table 2).

## 4. Discussion

The depth of preparation in discolored teeth for veneering depends on the severity of the discoloration and the position of the tooth. Therefore, the preparation depth of discolored teeth should extend to between 0.5 and 1 mm [23]. A change of one or two shades in the shade guide is generally possible to obtain with a thin laminate (0.3 mm thickness). However, greater changes in color shade require more invasive preparation [11]. Thus, 0.5 and 1 mm preparation depths were adopted in this study, which is aimed at evaluating the effect of bleaching before or after veneer preparation and the preparation depth on the final color of laminate veneers.

Light shades of ceramic are most commonly used in veneer construction, and the translucent ceramic veneer allows the assessment of the effect of the tooth substrate shade on final color of the laminate veneers [24], and as the cement shade has the ability to change the final color of the restoration to a significant extent [9], shade A1 HT (highly translucent) IPS e-max ceramic was used in the current study which was cemented with translucent luting cement, with the middle third of the crown of the teeth being selected for color change measurement because it is the most representative area in terms of color [25].

Since high concentrations of hydrogen peroxide are indicated when faster results are needed [4] and a maximal effect can be obtained after a single application [5], one session of in-office bleaching protocol was used to whiten the teeth and the bleaching produced a significant color change which is also consistent with a previous study [17]. The tooth whitening was performed by applying 35% hydrogen peroxide on the external surface of the teeth which has been activated by light; the light activates the agent by heat generation. Although activation by light is recommended by the manufacturer, it has been reported in some literature that light activation of in-office bleaching gel does not affect the bleaching efficacy regardless of hydrogen peroxide concentration [26]. A Δ*E* value of less than one is considered visible to a trained operator only, whereas a Δ*E* of 3.7 or more is clinically visible [27]. However, all color changes produced were all within the clinically visible range (Δ*E* > 3.7).

The effect of enamel thickness on the bleaching efficacy was obvious in this study. Although our results revealed no significant difference statistically between the BBP and BAP groups, the Δ*E* mean values of BAP were higher than BBP for either S1 or S2. After tooth preparation, hydrogen peroxide penetration was enhanced and recorded a higher Δ*E* mean value than bleaching before preparation. These results agree with Shin and Summitt's study, which found that bleaching of thinned enamel resulted in a significant color change, and that thick enamel can act as a strong barrier to vital bleaching [6]. The bleaching agent applied to the surface of enamel must, in order to bring about the whitening effect, penetrate sufficiently through the enamel layer to the dentine to alter the color of the dentine immediately below the enamel, as bleaching through the enamel does not penetrate the entire depth of the dentine [16]. In addition, the data from a previous study demonstrated that the presence of at least 0.5 mm of enamel reduced the bleaching efficacy, and the presence of aprismatic enamel resulted in less color change in comparison to the group without aprismatic enamel [7].

The thickness of the ceramic restoration also can influence the overall shade and produce a significant change in Δ*E*, which could be due to its translucency, as the thicker the ceramic, the less the translucency, with the consequence decrease in the diffused reflection effects of the underlying tooth substrate [8, 28]. This may explain the high Δ*E* mean value of S2 in all tested groups compared to S1 in our study; however, the difference was not significant. These findings are consistent with other studies that reported that ceramic veneer thickness affects its masking ability [12, 29, 30]. Moreover, the ability of a veneer to mask the underlying discolored tooth is affected by the type, thickness, shade, and opacity of the ceramic and the cement used [9, 10, 31].

In the BAP group, 0.5 mm thick ceramic veneer (S1) resulted in a Δ*E* value not statistically significantly different than those obtained for 1 mm thick ceramic veneer (S2) of the three tested groups (NB, BBP, and BAP), which is in agreement with previous studies [13, 29], as when the lightness of the substrate shade is increased, a higher Δ*E* value can be achieved from a thinner ceramic veneer combination.

In respect of the spectrophotometer results, Δ*L*∗ is often used in studies of tooth bleaching as it assesses the lightness of the tooth [16], and the ability of a veneer to produce a color change is due to its ability to alter *b*∗ [32]. Corresponding results were found in this study; in the NB group, placing a veneer restoration resulted in a little change in *L*∗ (lightness) and *a*∗ (red/green), but produced a shift in *b*∗ (yellow/blue) of around 17 units for S1 and 20 units for S2 as the translucency decreased and opacity increased. Meanwhile, in the BBP and BAP groups, for both thicknesses (S1 and S2), the results showed a change in *a*∗ with a greater change in *L*∗ and less change in *b*∗ compared to NB group; these results are in agreement with other studies that reported an increase in lightness (*L*∗) and decrease in chroma (*a*∗ and *b*∗) and increase in whiteness after bleaching [33]. The BAP group recorded the highest change in *L*∗ value, which could be due to the enhanced bleaching effect when placed in contact with the exposed deeper layer of enamel, whereas a lower change in *L*∗ value was recorded in the NB group, as increasing the thickness of the ceramic decreased its brightness, which may be explained by the fact that more light is absorbed and less is reflected with increasing ceramic thickness [34]. In all tested groups, a decrease in *a*∗ and *b*∗ values was observed. These results suggest a reduction in redness and yellowness which are consistent with the previous studies [16, 28].

The most common side effect of bleaching is tooth sensitivity especially with high concentration hydrogen peroxide [4, 35]. Although it has been stated in the recent literature that the same bleaching efficacy could be achieved with lower concentration hydrogen peroxide to a high concentration hydrogen peroxide with lower risk of bleaching sensitivity intensity, the ideal concentration that can produce maximum bleaching with the lowest bleaching sensitivity intensity has not been determined yet [36]. Clinically, this side effect may restrict the amount of whitening that can be achieved, and it may be even more severe when the enamel thickness is reduced; however, vital tooth bleaching after preparation might not be recommended clinically, due to the fact that aprismatic enamel or compact enamel removal by preparation increases the deleterious effect of the bleaching by enhancement of penetration of the free radicals into the pulp [37], resulting in higher tooth sensitivity or pulp degeneration. Another side effect of bleaching is reduction in bond strength to bleached enamel surface when performing cementation immediately after bleaching, due to the presence of residual free radicals [38]; however, a recent study by Cheng et al. has revealed that similar bond strength can be achieved between the bleached and unbleached enamels when removing 0.5 mm from the enamel surface [39].

Consequently, when teeth are severely stained to the point that routine bleaching procedure by application of the bleaching agent to the surface of the enamel has no effect and when veneering is intended to mask the discoloration, to reduce the amount of tooth destruction and preserve the dental tissue, it may be possible to whiten them by exposing the deeper enamel layer directly to the bleaching agent via veneer preparation without exposing dentine. The results of this study indicated that in cases of severely discolored teeth BAP has a similar effect of BBP on the final color of laminate veneers at different preparation depths, increasing the preparation depth does not influence the final color of laminate veneers, and one session of in-office bleaching after 0.5 mm veneer preparation depth can be as effective as 1.0 mm veneer preparation depth without bleaching in masking the underlying tooth discoloration. For any tooth whitening treatment, the need to carry out clinical and radiological check-ups must not be disregarded to ensure that patients do not suffer the possible negative effects that can occur for each treatment modality and to decide whether to deep cut or bleach.

Since the results obtained in this study are limited to an in vitro situation and nonvital premolar teeth have been used, the results could have been more realistic if anterior teeth had been used. However, their availability is limited, and moreover, vital teeth in an in vivo state may produce different results. Even though the artificial staining of the teeth was done by a method that has been used successfully in previous studies [16], the staining process in the mouth is more complex and involves multifactorial aetiology, which may result in different bleaching responses. Other factors that might influence this study's results are the bleaching type, concentration and application time, and ceramic shades, and types. Hence, further in vitro and in vivo studies are necessary to confirm these results and to provide further information about the aspects of bleaching after veneer preparation including bleaching sensitivity intensity and bonding strength in comparison to bleaching before preparation.

## 5. Conclusions

Despite the limitations mentioned in this study, it can be concluded that in cases of severe tooth discoloration one session of in-office bleaching before or after veneer preparation and the depth of preparation do not affect the color masking ability of laminate veneers. However, one session of in-office bleaching of the enamel surface after removal of 0.5 mm can compensate for 1 mm preparation depth without bleaching when the veneer is intended to mask underlying tooth discoloration in severely discolored teeth, which means that the depth of preparation can be reduced by about 0.5 mm.

## Figures and Tables

**Table 1:** Mean (SD) of color change (Δ*E*) and color coordinates (Δ*L*∗, Δ*a*∗, and Δ*b*∗).

Group		Δ*L*∗	Δ*a*∗	Δ*b*∗	Δ*E*
0.5 mm	NB	3.98 (1.6)^a^	-2.44 (0.5)^a^	-16.6 (2.9)^a^	17.38 (2.6)^a^
	BBP	9.19 (3.02)^b^	-2.72 (1.1)^a^	-14.3 (5.0)^a^	17.85 (3.5)^a^
	BAP	8.39 (4.7)^a,b^	-3.2 (1.5)^a^	-16.6 (5.5)^a^	19.9 (3.6)^a,b^

1.0 mm	NB	4.68 (3.3)^a,c^	-3.5 (1.1)^a^	-18.2 (4.5)^a^	19.6 (3.8)^a,b^
	BBP	8.57 (4.1)^c,b^	-2.87 (0.8)^a^	-17.6 (1.8)^a^	20.13 (2.4)^a,b^
	BAP	13.2 (2.4)^d,b^	-3.6 (0.9)^a^	-18.2 (3.3)^a^	22.99 (2.9)^b^

NB = nonbleaching; BBP = bleaching before preparation; BAP = bleaching after preparation; SD = standard deviation. ^a–d^Identical letters indicate no significant differences (*p* ≤ 0.05, Tukey's test).

**Table 2:** ANOVA test showing the significance among the tested groups.

		Sum of squares	df	Mean square	*F*	Sig.
Δ*L*∗	Between groups	562.199	5	112.440	10.025	0.0001
	Within groups	605.640	54	11.216		
	Total	1167.839	59			

Δ*a*∗	Between groups	10.163	5	2.033	1.861	0.116
	Within groups	58.967	54	1.092		
	Total	69.130	59			

Δ*b*∗	Between groups	108.406	5	21.681	1.289	0.282
	Within groups	908.323	54	16.821		
	Total	1016.729	59			

Δ*E*	Between groups	198.698	5	39.740	3.880	0.004
	Within groups	553.084	54	10.242		
	Total	751.782	59			

## Data Availability

Data could be provided upon request from Dr. Gollshang Ahmad Mhammed Dalloo (gollshang.mhammed@univsul.edu.iq).

## References

[1] Sulieman M. (2005). An overview of bleaching techniques: 3. In-surgery or power bleaching. *Dental Update*.

[2] Dubal R., Porter R. W. (2018). An update on discoloured teeth and bleaching part 1: the aetiology and diagnosis of discoloured teeth. *Dental Update*.

[3] Kose C., Reis A., Baratieri L. N., Loguercio A. D. (2011). Clinical effects of at-home bleaching along with desensitizing agent application. *American Journal of Dentistry*.

[4] Borges A. B., Zanatta R. F., Barros A. C. S. M., Silva L. C., Pucci C. R., Torres C. R. G. (2015). Effect of hydrogen peroxide concentration on enamel color and microhardness. *Operative Dentistry*.

[5] Amengual-Lorenzo J., Montiel-Company J. M., Labaig-Rueda C., Agustín-Panadero R., Solá-Ruíz M. F., Peydro-Herrero M. (2020). Combined vital tooth whitening: effect of number of in-office sessions on the duration of home whitening. A randomized clinical trial. *Applied Sciences*.

[6] Shin D., Summitt J. B. (2002). The whitening effect of bleaching agents on tetracycline-stained rat teeth. *Operative Dentistry*.

[7] do Carmo Públio J., D’Arce M. B. F., Catelan A. (2016). Influence of enamel thickness on bleaching efficacy: an in-depth color analysis. *The Open Dentistry Journal*.

[8] Xing W., Chen X., Ren D., Zhan K., Wang Y. (2017). The effect of ceramic thickness and resin cement shades on the color matching of ceramic veneers in discolored teeth. *Odontology*.

[9] Montero J., Gómez‐Polo C. (2016). Effect of ceramic thickness and cement shade on the final shade after bonding using the 3D master system: a laboratory study. *Clinical and Experimental Dental Research*.

[10] Begum Z., Chheda P., Shruthi C. S., Sonika R. (2014). Effect of ceramic thickness and luting agent shade on the color masking ability of laminate veneers. *The Journal of Indian Prosthodontic Society*.

[11] Coachman C., Gurel G., Calamita M., Morimoto S., Paolucci B., Sesma N. (2014). The influence of tooth color on preparation design for laminate veneers from a minimally invasive perspective: case report. *The International Journal of Periodontics & Restorative Dentistry*.

[12] Omar H., Atta O., El-Mowafy O., Khan S. A. (2010). Effect of CAD-CAM porcelain veneers thickness on their cemented color. *Journal of Dentistry*.

[13] Faus-Matoses V., Faus-Matoses I., Ruiz-Bell E., Faus-Llacer V. J. (2017). Severe tetracycline dental discoloration: restoration with conventional feldspathic ceramic veneers. A clinical report. *Journal of Clinical and Experimental Dentistry*.

[14] Blunck U., Fischer S., Hajtó J., Frei S., Frankenberger R. (2020). Ceramic laminate veneers: effect of preparation design and ceramic thickness on fracture resistance and marginal quality in vitro. *Clinical Oral Investigations*.

[15] Yu H., Zhao Y., Li J. (2019). Minimal invasive microscopic tooth preparation in esthetic restoration: a specialist consensus. *International Journal of Oral Science*.

[16] Sulieman M., Addy M., Rees J. S. (2003). Development and evaluation of a method in vitro to study the effectiveness of tooth bleaching. *Journal of Dentistry*.

[17] Sulieman M., Addy M., Macdonald E., Rees J. S. (2005). The bleaching depth of a 35% hydrogen peroxide based in-office product: a study in vitro. *Journal of Dentistry*.

[18] Daouahi N. (2020). Management of dental fluorosis with bleaching and ceramic veneers : clinical report. *American Journal of Dentistry and Oral Care*.

[19] Mushashe A. M., Coelho B. S., Garcia P. (2018). Effect of different bleaching protocols on whitening efficiency and enamel superficial microhardness. *Journal of Clinical and Experimental Dentistry*.

[20] Castellanos M., Delgado A. J., Sinhoreti M. A. C. (2019). Effect of thickness of ceramic veneers on color stability and bond strength of resin luting cements containing alternative photoinitiators. *The Journal of Adhesive Dentistry*.

[21] Mena-Serrano A. P., Parreiras S. O., do Nascimento E. M. S. (2015). Effects of the concentration and composition of in-office bleaching gels on hydrogen peroxide penetration into the pulp chamber. *Operative Dentistry*.

[22] Roderjan D. A., Stanislawczuk R., Hebling J., de Souza Costa C. A., Reis A., Loguercio A. D. (2015). Response of human pulps to different in-office bleaching techniques: preliminary findings. *Brazilian Dental Journal*.

[23] Petricevic N. P. I. (2017). Procelain veneers and zircon-porcelain crowns for the ethic treatment of servely discoloured anterior teeth. *JSM Oro Facial Surgeries*.

[24] Jankar A. S. (2015). Spectrophotometric study of the effect of luting agents on the resultant shade of ceramic veneers: an invitro study. *Journal of Clinical and Diagnostic Research*.

[25] Goodkind R. J., Schwabacher W. B. (1987). Use of a fiber-optic colorimeter for in vivo color measurements of 2830 anterior teeth. *The Journal of Prosthetic Dentistry*.

[26] Maran B. M., Burey A., de Paris Matos T., Loguercio A. D., Reis A. (2018). In-office dental bleaching with light vs. without light: a systematic review and meta-analysis. *Journal of Dentistry*.

[27] Seghi R. R., Hewlett E. R., Kim J. (1989). Visual and instrumental colorimetric assessments of small color differences on translucent dental porcelain. *Journal of Dental Research*.

[28] Tamam E., Güngör M., Nemli S. (2020). How are the color parameters of a CAD/CAM feldspathic ceramic of the material affected by its thickness, shade, and color of the substructure?. *Nigerian Journal of Clinical Practice*.

[29] Sari T., Ural C., Yüzbasioglu E., Duran I., Cengiz S., Kavut I. (2018). Color match of a feldspathic ceramic CAD-CAM material for ultrathin laminate veneers as a function of substrate shade, restoration color, and thickness. *The Journal of Prosthetic Dentistry*.

[30] El-Anwar M. I., Kandil B. S. M., Hamdy A. M., Aboelfadl A. K. (2019). Effect of ceramic translucency and luting cement shade on the color masking ability of laminate veneers. *Dental Research Journal*.

[31] Pires L. A., Novais P. M. R., Araújo V. D., Pegoraro L. F. (2017). Effects of the type and thickness of ceramic, substrate, and cement on the optical color of a lithium disilicate ceramic. *The Journal of Prosthetic Dentistry*.

[32] Griffiths C. E., Bailey J. R., Jarad F. D., Youngson C. C. (2008). An investigation into most effective method of treating stained teeth: an in vitro study. *Journal of Dentistry*.

[33] Pan Q., Westland S. (2018). Tooth color and whitening - digital technologies. *Journal of Dentistry*.

[34] Giti R., Hojati S. A. (2018). Effect of varying thickness and number of coloring liquid applications on the color of anatomic contour monolithic zirconia ceramics. *Journal of Dentistry*.

[35] Lima S. N. L., Ribeiro I. S., Grisotto M. A. (2018). Evaluation of several clinical parameters after bleaching with hydrogen peroxide at different concentrations: a randomized clinical trial. *Journal of Dentistry*.

[36] Maran B. M., de Paris Matos T., dos Santos de Castro A. (2020). In-office bleaching with _low_ / _medium_ vs. _high_ concentrate hydrogen peroxide: a systematic review and meta-analysis. *Journal of Dentistry*.

[37] de Oliveira Duque C. C., Soares D. G., Basso F. G., Hebling J., de Souza Costa C. A. (2017). Influence of enamel/dentin thickness on the toxic and esthetic effects of experimental in-office bleaching protocols. *Clinical Oral Investigations*.

[38] Briso A. L., Toseto R. M., Rahal V., Dos Santos P. H., Ambrosano G. M. (2012). Effect of sodium ascorbate on tag formation in bleached enamel. *The Journal of Adhesive Dentistry*.

[39] Cheng Y.-l., Musonda J., Cheng H., Attin T., Zheng M., Yu H. (2019). Effect of surface removal following bleaching on the bond strength of enamel. *BMC Oral Health*.

